# Draft genome sequence of *Ligilactobacillus agilis* strain SY88 isolated from yogurt in Rajshahi, Bangladesh

**DOI:** 10.1128/mra.00700-26

**Published:** 2026-07-29

**Authors:** Nayeema Talukder Ema, Ayan Goshwami, Anika Tasnim, Amit Kumar Dey, Dipa Rani Roy, Sabbir Ahmed, Md. Ahashan Habib, Md. Salim Khan

**Affiliations:** 1 Institute of Food Science and Technology, Bangladesh Council of Scientific and Industrial Research (BCSIR) 130051, Dhaka, Bangladesh; 2 BCSIR Rajshahi Laboratories, BCSIR 130051 , Rajshahi, Bangladesh; 3 Biomedical and Toxicological Research Institute (BTRI), BCSIR 130051 , Dhaka, Bangladesh; 4 Institute of National Analytical Research and Service, BCSIR 130051 , Dhaka, Bangladesh; Nanchang University, Nanchang, Jiangxi, China

**Keywords:** *Ligilactobacillus agilis*, lactic acid bacteria, whole-genome sequencing, fermentation

## Abstract

*Ligilactobacillus agilis* is a lactic acid bacterium isolated from traditional yogurt in Rajshahi. Here, we report the draft whole-genome sequence of *Ligilactobacillus agilis SY88*, with a genome size of 1,998,117 bp and GC content of 41.86%. This genome sequence expands the genomic resources for dairy-associated lactic acid bacterial species.

## ANNOUNCEMENT

Lactic acid bacteria (LAB) are a heterogeneous group of bacteria having a prominent role in food fermentation, and most are generally recognized as safe (GRAS) (1, 2). *Ligilactobacillus agilis* is an LAB frequently isolated from pigeon crops, the gastrointestinal tract of birds and humans, and various fermented foods (3). In this study, *Ligilactobacillus agilis* SY88 was isolated from traditionally fermented yogurt, obtained from a local confectionery shop (24.36784°N, 88.64497°E) in Rajshahi, Bangladesh, in 2025. A 10 g yogurt sample was serially diluted, plated onto de Man-Rogosa-Sharpe (MRS) agar plates, and incubated anaerobically in an anaerobic jar at 37°C for 48 h. A pure culture was obtained with repeated streaking, then identified and stored with 30% glycerol at −80°C for further analysis.

For whole-genome sequence (WGS) analysis, an MRS agar plate containing a pure culture of *Ligilactobacillus agilis* SY88 was submitted to the icddr,b Genome Center (iGC), International Center for Diarrhoeal Disease Research, Mohakhali, Dhaka, Bangladesh. Genomic DNA extraction was performed according to the manufacturer’s instructions using the Wizard Genomic DNA Purification Kit (Cat. A1620, Promega, Madison, WI, USA). Library preparation was done by Illumina DNA Prep (Illumina, San Diego, CA, USA) according to the manufacturer’s instructions. Sequencing was performed on the Illumina NextSeq 2000 platform using the 2 × 150 bp paired-end read length sequencing protocol. The quality of the raw reads was analyzed using FastQC v0.12.1 (4) and filtered with FastP v0.23.4 (5). The sequencing data were assembled by *de novo* assembly using SPAdes v4.0.0 (6). Genome completeness and contamination were assessed through CheckM2 v1.1 (7). The comprehensive genome analysis services provided by BV-BRC v3.58.4 (8) were used for genome annotation. The average nucleotide identity (ANI) analysis was done using FastANI v1.34 (9), which showed 97.3% identity with *Ligilactobacillus agilis* DSM 20509 (NCBI RefSeq assembly: GCF_001436215.1). Then functional annotation and subsystem categorization were performed by Rapid Annotations using Subsystems Technology (RAST) via SEED Viewer version 2.0 (10). Virulence factors were screened using the VFDB Database (11) with ABRicate v1.4 (12) and antibiotic resistance genes by using the Resistance Gene Identifier (RGI) web server v6.0.5 against the Comprehensive Antibiotic Resistance Database (CARD) v4.0.1 with default parameters (13). Presence of plasmids and probability of human pathogens were identified by PlasmidFinder v2.1 (14) and PathogenFinder v1.1 (15). The bioinformatic analyses were done using default parameters of each software tool unless otherwise stated.

The genome contained 121 contigs, totaling a genome size of 1,998,117 bp and a GC content of 41.86%. (Table 1). Subsystem category distribution using RAST identified 205 subsystems. Functional annotation revealed genes associated with carbohydrate metabolism and fermentation, including lactose (*lacS* and l*acZ*), galactose (*galK* and *galT*), and glycogen (*glgA*, *glgB*, *glgC*, and *glgP*). No significant toxin gene was found. No plasmid-associated sequence was detected in the genome. Furthermore, the strain was predicted as a non-human pathogen with a low pathogenic probability score of 0.272.

**TABLE 1:** Summary of the genomic features of *Ligilactobacillus agilis* SY88

Parameters	*Ligilactobacillus agilis* SY88
Total number of reads	996,632 reads
Contigs	121
Genome size (bp)	1,998,117 bp
GC content (%)	41.86
Contig *L*_50_	18
Contig *N*_50_	41,842
Coverage (×)	68.42
Completeness (%)	99.8
CDS	2,010
tRNAs	54
rRNAs	5
Hypothetical proteins	624
Proteins with functional assignments	1,386
Proteins with EC number assignments	530
Proteins with GO assignments	442
Proteins with pathway assignments	371
Antibiotic-resistant genes	2

## Data Availability

This Whole Genome Shotgun project has been deposited at DDBJ/ENA/GenBank under accession number JBXHAX000000000, BioProject accession number PRJNA1455380, and BioSample accession number SAMN57363931. The version described in this paper is version JBXHAX010000000. The raw sequencing reads have been deposited in the NCBI Sequence Read Archive under accession number SRR39086265.
